# Metabolic Flux Adaptations During GLP-1 Receptor Agonist Therapy: Redox Implications

**DOI:** 10.1007/s13679-026-00709-4

**Published:** 2026-04-01

**Authors:** Jaewang Lee, Ji Hyeon You, Hyun Jin Park, Jong-Lyel Roh, Sun Ha Paek

**Affiliations:** 1 Logsynk Ltd, Seoul, Republic of Korea; 2 Department of Otorhinolaryngology-Head and Neck Surgery, CHA Bundang Medical Center, CHA University, Seongnam, Republic of Korea; 3 Department of Neurosurgery, Seoul National University College of Medicine, Seoul National University Hospital, Seoul, Republic of Korea; 4 Seoul National University Cancer Research Institute, Seoul National University College of Medicine, Seoul, Republic of Korea; 5 Interdisciplinary Program in Cancer Biology, Seoul National University College of Medicine, Seoul, Republic of Korea; 6 Department of Biomedical Science, General Graduate School, CHA University, Pocheon, Republic of Korea; 7 Hypoxia/Ischemia Disease Institute, Cancer Research Institute, Seoul National University College of Medicine, Seoul, Republic of Korea; 8 Advanced Institutes of Convergence Technology, Seoul National University, Suwon, Republic of Korea

**Keywords:** GLP-1 receptor agonists, Metabolic flux, Energy metabolism, NAD⁺ metabolism, Nutritional metabolism

## Abstract

**Purpose of Review:**

GLP-1 receptor agonists (GLP-1RAs) have become central therapies for obesity and metabolic disease. Although these agents produce substantial and sustained weight reduction, emerging clinical observations suggest that chronic therapy may be accompanied by changes in lean mass trajectory, reduced protein intake, and micronutrient insufficiency. This review examines metabolic adaptations during GLP-1RA therapy and their implications for nutritional and redox homeostasis.

**Recent Findings:**

GLP-1RA therapy maintains receptor signaling while reducing nutrient inflow, creating a physiological state of chronic energy flux constriction. Under these conditions, systemic metabolism increasingly relies on lipid-derived substrates and elevated oxidative throughput. When oxidative demand approaches the regenerative capacity of nicotinamide adenine dinucleotide (NAD⁺)/ nicotinamide adenine dinucleotide phosphate (NADPH; reduced form)-dependent buffering systems, several constraint-sensitive metabolic processes—including NAD⁺ partitioning, amino acid–dependent antioxidant–anabolic coupling, micronutrient-dependent enzymatic activity, and bile acid–mediated absorption—may become functionally limiting.

**Summary:**

Integrating emerging mechanistic and clinical observations, this review synthesizes current evidence from a metabolic flux perspective. This approach highlights how nutritional substrates and metabolic cofactors may influence physiological resilience during sustained pharmacological weight reduction. Clarifying these interactions may inform clinical monitoring strategies and nutritional management during long-term GLP-1RA therapy.

## Introduction

Obesity, defined as a body mass index (BMI) ≥ 30 kg/m², represents a major global health burden [1–3]. In 2022, global pooled analyses estimated that approximately one in eight individuals worldwide were living with obesity, encompassing 890 million adults and 160 million children and adolescents [2]. These epidemiological trends have accelerated the development of pharmacological strategies capable of inducing sustained weight reduction. Glucagon-like peptide-1 receptor agonists (GLP-1RAs) have emerged as highly effective anti-obesity therapies, producing weight loss of approximately 5–20% across clinical trials. Therapeutic innovation now includes dual and triple incretin receptor agonists [4–7] as well as orally bioavailable small-molecule formulations [8, 9], reflecting ongoing efforts to enhance pharmacodynamic durability. These agents enable sustained GLP-1 receptor engagement and thereby modulate appetite regulation, gastric emptying, and insulin–glucagon signaling.

However, GLP-1RA therapy also induces persistent hypophagia and altered nutrient delivery kinetics, leading to chronically reduced systemic substrate inflow. Recognized adverse effects include cholelithiasis, gastrointestinal disturbances, and reductions in lean mass [10–12]. In addition, long-term therapeutic responses may attenuate over time [13], suggesting adaptive responses within incretin signaling networks. Beyond these clinically recognized effects, sustained caloric restriction during pharmacological weight reduction represents a chronic constraint on systemic energy flux.

Despite these therapeutic advances, the metabolic consequences of sustained pharmacologically induced hypophagia remain incompletely characterized. Weight loss alone does not fully capture the biochemical adaptations occurring during prolonged GLP-1RA therapy, particularly those related to substrate redistribution, redox balance, and micronutrient-dependent metabolic processes. Energy restriction requires coordinated metabolic reorganization involving hepatic glucose output, adipose tissue lipolysis, skeletal muscle protein turnover, and mitochondrial fuel selection. Increased reliance on fatty acid oxidation (FAO) alters mitochondrial electron flux and redox cofactor dynamics, influencing both anabolic–catabolic balance and oxidative buffering capacity. Although GLP-1RAs confer anti-inflammatory and glycemic benefits, persistent reductions in nutrient inflow and delayed gastric emptying may limit the availability of amino acids and micronutrients required for tissue maintenance and redox homeostasis. In individuals with obesity—who frequently exhibit baseline micronutrient perturbations and altered iron metabolism [14–17]—such adaptations may have amplified metabolic consequences. Taken together, GLP-1RA therapy can be viewed as a sustained remodeling of systemic energy flux and substrate prioritization. The durability and safety of pharmacologically induced weight loss may therefore depend on whether biochemical adaptation—particularly protein sufficiency, micronutrient-dependent enzymatic throughput, and redox currency regeneration—remains proportionate to oxidative demand.

In this review, we examine GLP-1RA therapy from a metabolism-centered perspective, integrating incretin signaling with substrate redistribution, mitochondrial metabolic throughput, and tissue-specific energetic constraints. We synthesize current mechanistic and clinical observations within a framework of energy flux constriction and substrate proportionality, highlighting metabolic factors that may influence nutritional stability during sustained pharmacological weight reduction.

## GLP-1RAs: Structure, Function, and Therapeutic Scope

### Molecular Basis: Endogenous GLP-1, Receptor Pharmacology, and Drug Engineering

Endogenous GLP-1 is a 30–amino-acid peptide generated by enzymatic processing of proglucagon in intestinal L-cells, pancreatic islet α-cells, and neurons of the nucleus of the solitary tract [10, 18]. Its biological effects are mediated through the GLP-1 receptor (GLP-1R), a class B G protein–coupled receptor with seven transmembrane domains [19]. Upon ligand binding, GLP-1R activation enhances glucose-dependent insulin secretion, suppresses glucagon release, delays gastric emptying, and reduces appetite, collectively improving glycemic control and promoting clinically meaningful weight reduction [19]. However, native GLP-1 is rapidly degraded by dipeptidyl peptidase-4 (DPP-4) and cleared renally, resulting in a plasma half-life of approximately 2–3 min [20, 21]. This pharmacokinetic limitation prompted development of GLP-1RAs that maintain receptor engagement while resisting enzymatic degradation.

The modern GLP-1RA class evolved following identification of exendin-4, a GLP-1 analogue originally isolated from Gila monster venom [22]. Contemporary therapeutic platforms include single agonists, dual and triple incretin receptor agonists, and emerging small-molecule agonists. Molecular durability is achieved through peptide engineering strategies that (i) confer resistance to DPP-4 cleavage, typically via N-terminal modification, and/or (ii) extend systemic exposure through fatty-acid acylation, which enhances albumin binding and slows renal clearance, thereby prolonging receptor activation [18].

Consistent with these principles, clinically approved anti-obesity GLP-1RAs include liraglutide, semaglutide, and tirzepatide [23–31], while beinaglutide is only approved in China [32]. A defining pharmacologic feature of these agents is prolonged receptor occupancy, which distinguishes therapeutic GLP-1R signaling from its endogenous, pulsatile counterpart.

Importantly, the metabolic consequences of GLP-1RA therapy arise not only from appetite suppression but also from sustained GLP-1R signaling under conditions of chronically reduced nutrient inflow. This combination establishes the upstream context for the energy flux constriction and systemic metabolic adaptations discussed in subsequent sections.

### Therapeutic Scope and the Translational “Mismatch” Problem

Clinically, GLP-1RAs are established therapies for obesity and type 2 diabetes (T2D), with expanding indications and ongoing investigation across multiple organ systems. Reported or emerging application areas include joint disorders [33, 34], psychotic and neurocognitive conditions [35], cardiovascular disease [36], and gastrointestinal disorders [37–39]. Despite this broad therapeutic interest, certain domains—particularly skeletal muscle biology and neurodegeneration—illustrate a recurring translational mismatch between mechanistic signaling effects and clinical functional outcomes.

First, body composition responses remain debated. Clinical studies have raised concerns regarding reductions in lean body mass during GLP-1RA therapy [40], whereas preclinical studies report improvements in muscle mass and strength [41–43]. This divergence suggests that net muscle outcomes may depend on contextual factors, including energy and protein availability, physical activity, and baseline sarcopenia risk—variables that are rarely examined mechanistically alongside pharmacological exposure.

Second, neurodegenerative indications highlight a similar gap between mechanistic promise and clinical outcomes. Although preclinical studies suggest that GLP-1RAs may attenuate Alzheimer’s disease (AD) progression [44, 45], phase III semaglutide trials (NCT04777396, NCT04777409) did not demonstrate clinical benefit. Likewise, despite encouraging preclinical findings in Parkinson’s disease (PD) [46], a phase III exenatide trial failed to slow motor decline [47]. Together, these observations suggest that receptor-level signaling and anti-inflammatory effects alone may be insufficient to produce functional improvement in energy-demanding tissues such as skeletal muscle and brain, where substrate turnover and mitochondrial metabolic throughput are critical determinants of tissue function.

These inconsistencies highlight the importance of a substrate-centered metabolic perspective. While GLP-1RAs improve inflammatory tone and metabolic signaling, therapy simultaneously reduces nutrient intake and alters gastrointestinal nutrient delivery [20, 21, 48]. Consequently, tissue-level outcomes may depend not only on receptor activation but also on the availability of metabolic substrates required to sustain bioenergetic and redox balance under conditions of reduced nutrient inflow. This perspective provides the basis for subsequent sections examining nicotinamide adenine dinucleotide (phosphate) (NAD(P)H; reduced form) redox currency, amino acid–dependent antioxidant coupling, micronutrient-dependent enzymatic processes, and membrane oxidative resilience.

### Summary: What Sect. 2 Establishes

GLP-1RAs are engineered to overcome the rapid degradation of native GLP-1 through DPP-4–resistant modifications and exposure-extending strategies such as fatty-acid acylation, enabling sustained receptor occupancy. While this pharmacology underlies therapeutic benefit in obesity and T2D, it simultaneously establishes conditions of persistently reduced nutrient inflow. These considerations suggest that GLP-1RA biology is best interpreted within a broader metabolic context in which substrate availability, redox buffering capacity, and tissue-specific energetic constraints shape systemic adaptation to sustained pharmacological weight reduction.

## GLP-1RA Therapy as a Chronic Flux Perturbation

### System-Level Energy Flux Reorganization Under Weight Loss Therapy

Most weight-loss interventions—including exercise [49], calorie restriction [50], cold exposure [51], and selected antidiabetic agents [10, 52]—ultimately increase reliance on FAO, which supplies acetyl-CoA to sustain tricarboxylic acid (TCA) cycle activity and electron transport chain (ETC)–coupled adenosine triphosphate (ATP) production. GLP-1RA therapy converges on this oxidative axis but uniquely couples it with sustained hypophagia and reduced exogenous nutrient inflow. By suppressing appetite and delaying gastric emptying, GLP-1RAs chronically reduce nutrient delivery from the diet [53]. Under these conditions, systemic metabolism must reprioritize substrate utilization. Reduced carbohydrate availability constrains glycolytic flux and increases dependence on lipid-derived substrates.

Enhanced FAO increases mitochondrial electron delivery to the ETC and alters redox cofactor cycling, particularly nicotinamide adenine dinucleotide (NAD⁺; oxidized form)/nicotinamide adenine dinucleotide (NADH; reduced form) and nicotinamide adenine dinucleotide phosphate (NADP⁺; oxidized form)/nicotinamide adenine dinucleotide phosphate (NADPH; reduced form) dynamics [54]. At the same time, AMP-activated protein kinase (AMPK) suppresses ATP-intensive anabolic programs to preserve energetic stability [52]. GLP-1RAs can also enhance glycolytic and oxidative pathways via AMPK activation, thereby limiting ectopic lipid accumulation and lipotoxic stress [55, 56]. Concurrently, improved insulin sensitivity suppresses adipose tissue lipolysis and reduces circulating free fatty acid concentrations [57]. GLP-1RA therapy has also been shown to improve postprandial lipid metabolism in humans, consistent with context-dependent modulation of systemic lipid flux [58].

This bidirectional regulation reflects metabolic state dependence. In nutrient-replete states, GLP-1–induced insulin signaling suppresses adipose lipolysis, whereas during reduced caloric intake and relative glucose scarcity, lipid substrates become the dominant oxidative fuel [59, 60]. Accordingly, GLP-1RA therapy dynamically reorganizes substrate hierarchy within a background of constrained energy inflow. The defining feature of this metabolic state is chronic energy flux constriction—a persistent reduction in nutrient delivery coupled with maintained or redistributed oxidative demand.

### Oxidative Throughput and Peroxidation Liability

Enhanced FAO supports weight reduction and reduces ectopic lipid accumulation [54]. However, when fatty acid delivery exceeds mitochondrial oxidative and redox-buffering capacity, increased electron transport pressure can elevate reactive oxygen species (ROS) production, promote endoplasmic reticulum stress, and increase susceptibility to lipid peroxidation [54, 61–63]. Beyond absolute oxidative rate, mitochondrial resilience depends on spare respiratory capacity—the difference between basal and maximal electron transport flux—which reflects the ability to accommodate additional energetic demand without redox disequilibrium [64, 65]. Under sustained reductions in nutrient inflow, metabolic reserve may narrow as reduced substrate availability limits mitochondrial redox regeneration and constrains the capacity to match oxidative flux with regenerative turnover. In this context, even moderate increases in oxidative demand can favor conditions that increase mitochondrial electron leakage and ROS formation, particularly when respiratory reserve and redox-buffering capacity are reduced. Such vulnerability does not necessarily indicate intrinsic mitochondrial dysfunction but may instead reflect reduced metabolic reserve relative to substrate-supported redox regeneration. This vulnerability is often amplified in obesity, where baseline oxidative tone is elevated and polyunsaturated fatty acid (PUFA)-enriched membrane phospholipids provide oxidation-susceptible substrates [66–68].

Within this metabolic context, GLP-1RA therapy may create conditions in which reliance on lipid-derived substrates increases the functional importance of redox-buffering systems that maintain oxidative stability.

Mobilization of fatty acids containing PUFA moieties can further increase susceptibility to lipid peroxidation [69, 70]. In addition, labile iron pools can catalyze lipid radical propagation through Fenton-type reactions, further amplifying membrane lipid peroxidation [71, 72]. Under sustained GLP-1RA–induced hypophagia, oxidative stability may become increasingly dependent on adequate antioxidant and redox-buffering capacity. Obesity-associated oxidative stress increases glutathione (GSH) turnover, potentially elevating demand for amino acid precursors required for antioxidant defense [73, 74].

**Fig. 1:**
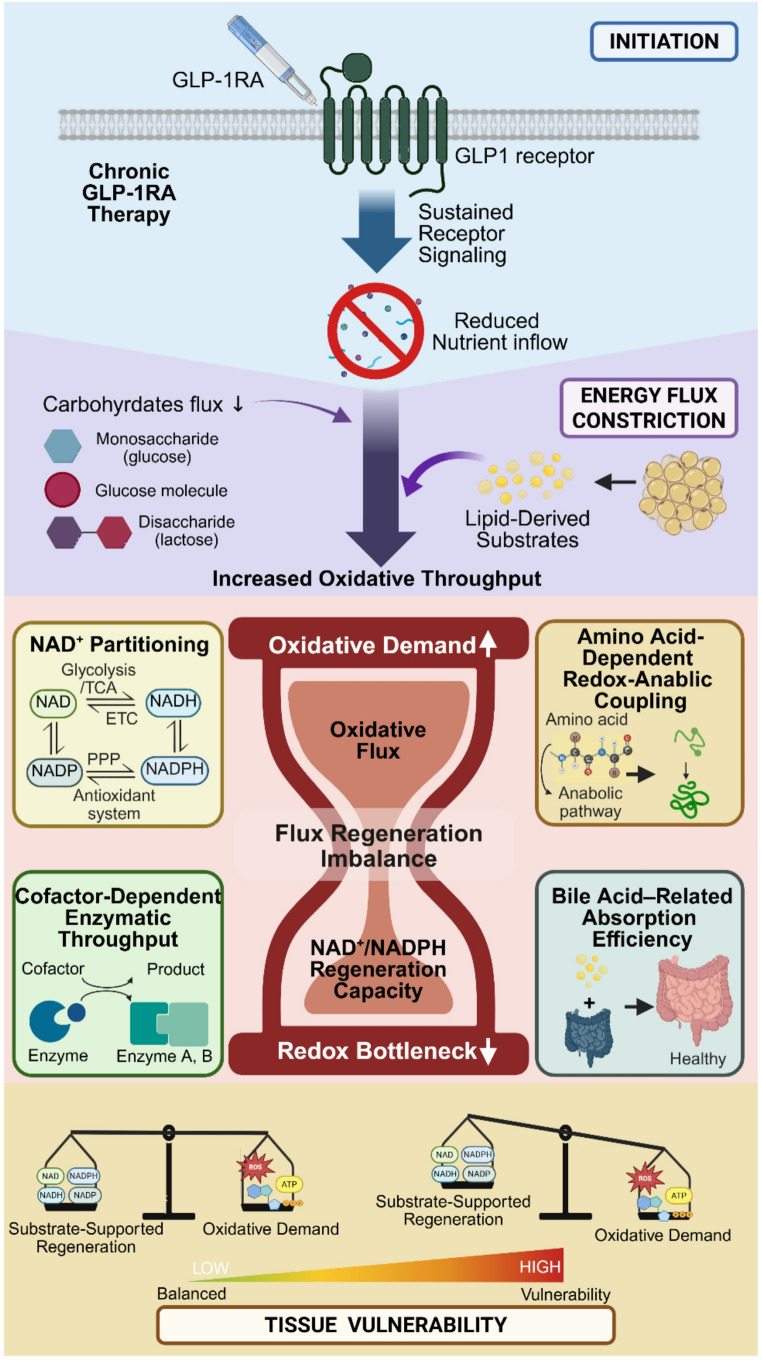
Chronic GLP-1RA therapy induces sustained receptor signaling while reducing nutrient inflow, generating a state of energy flux constriction. Under these conditions metabolism increasingly relies on lipid-derived substrates and oxidative throughput. When oxidative demand approaches the regenerative capacity of NAD⁺/NADPH-dependent buffering systems, a conditional “*redox bottleneck*” may emerge. Constraint-sensitive modules—including NAD⁺ partitioning, amino acid–dependent redox–anabolic coupling, cofactor-dependent enzymatic throughput, and bile acid–related absorption—determine tissue-level vulnerability. Abbreviations: ATP, adenosine triphosphate; ETC, electron transport chain; GLP-1RA, glucagon-like peptide-1 receptor agonist; NAD(P), nicotinamide adenine dinucleotide (phosphate) (oxidized form); NAD(P)H, nicotinamide adenine dinucleotide (phosphate) (reduced form); PPP, pentose phosphate pathway; ROS, reactive oxygen species; TCA, tricarboxylic acid cycle. Created in BioRender. YOU, J. (2026) https://BioRender.com/3you93u

Accordingly, the metabolic consequences of GLP-1RA therapy cannot be inferred solely from improvements in inflammatory tone or glycemic control. Clinical studies reporting variability in lean mass trajectories and dietary intake during GLP-1RA–associated weight loss [40, 75] suggest that adaptive stability may depend not only on receptor-mediated signaling but also on substrate availability during sustained hypophagia. When oxidative throughput approaches or exceeds regenerative redox-buffering capacity under constrained substrate inflow, a rate-limiting metabolic state can be conceptualized—referred to here as a “*redox bottleneck*” as discussed in Sect. 5 (Fig. 1).

### Transition to Metabolic Constraint

Collectively, GLP-1RA therapy is characterized by persistent reductions in nutrient inflow, increased reliance on lipid-derived substrates, suppression of anabolic pathways through AMPK signaling, and context-dependent modulation of adipose tissue lipolysis. These coordinated adaptations enable therapeutic weight reduction.

However, under sustained energy flux constriction, metabolic stability increasingly depends on substrate availability rather than signaling sufficiency alone. The balance between oxidative demand and redox buffering capacity—as well as between catabolic drive and anabolic substrate support—may therefore become an important determinant of tissue resilience and the durability of pharmacologically induced weight loss.

This flux-centered perspective provides a framework for identifying metabolic constraint nodes that may emerge during prolonged GLP-1RA therapy. These nodes are not intrinsically pathological but may become clinically relevant when oxidative demand and substrate availability diverge.

## Vulnerability Nodes Under Chronic Flux Constriction

Chronic reductions in nutrient inflow during GLP-1RA therapy do not simply alter energy balance but reshape the metabolic boundary conditions governing systemic homeostasis. When oxidative throughput remains sustained while substrate availability declines, discrete rate-limiting biochemical nodes may emerge [76, 77]. Adequate amino acid availability is required to sustain protein synthesis, antioxidant synthesis, and metabolic homeostasis, and reductions in amino acid supply may therefore influence the capacity of redox-buffering systems that maintain oxidative stability because glutathione synthesis depends on cysteine and related amino acid precursors [78, 79]. These constraint-sensitive processes may influence long-term tissue resilience beyond short-term weight-centered outcomes.

### Redox Throughput vs. Antioxidant Substrate Availability

GLP-1RAs demonstrate anti-inflammatory [80] and antioxidant-associated effects [81] across both preclinical and clinical studies, although findings are not entirely consistent [82]. These improvements are generally attributed to enhanced glycemic control and reduced inflammatory signaling rather than to expansion of substrate-dependent antioxidant buffering capacity. Cellular antioxidant systems rely on dynamic cycling of NAD⁺/NADH and NADP⁺/NADPH, glutathione turnover, and cofactor-dependent enzymatic reactions [83, 84]. Impairment of these redox currency systems compromises both antioxidant defense and mitochondrial bioenergetic stability [83]. Although clinical studies have not demonstrated overt systemic depletion of NAD(P)⁺ during GLP-1RA therapy, sustained reductions in nutrient inflow may influence precursor availability and turnover kinetics, particularly in metabolically stressed populations.

The cholesterol–mevalonate pathway illustrates this substrate dependence. Beyond structural membrane functions, this pathway supports biosynthesis of coenzyme Q10 (CoQ10) and glutathione peroxidase 4 (GPX4), both of which contribute to suppression of lipid peroxidation [67]. Since GLP-1RAs reduce circulating cholesterol and alter systemic lipid flux, downstream effects on membrane-associated redox buffering are biologically plausible, although direct clinical evidence remains limited. This distinction highlights the importance of differentiating signaling-mediated antioxidant effects from substrate-dependent determinants of membrane resilience.

During sustained hypophagia, alignment between oxidative demand and redox substrate availability may therefore become increasingly relevant. Although direct clinical evidence of systemic NAD(P)⁺ depletion during GLP-1RA therapy is lacking, experimental and observational studies suggest that NAD⁺ turnover and micronutrient status can be altered in obesity and inflammatory states [83, 85], supporting the biological plausibility of substrate-sensitive vulnerability. Caloric restriction has also been shown to alter circulating amino acid profiles and protein turnover dynamics [86, 87], further influencing systemic metabolic regulation under conditions of reduced nutrient inflow.

### Lean Mass Vulnerability and Anabolic Suppression

GLP-1RA therapy is frequently associated with reductions in lean body mass in clinical studies [40], although preclinical experiments have reported improvements in muscle function and mass under certain conditions [41–43]. Since GLP-1RA therapy reduces energy intake primarily through appetite suppression, and observational studies suggest that dietary protein intake may also decline during treatment [75]. This divergence suggests that skeletal muscle outcomes may depend on metabolic and behavioral context.

Under conditions of reduced caloric intake, AMPK-mediated suppression of anabolic pathways, combined with diminished amino acid availability, may constrain mechanistic target of rapamycin (mTOR) signaling and translational capacity [52, 88]. While adipose tissue reduction is therapeutically desirable, disproportionate loss of lean mass may lower resting energy expenditure, impair functional capacity, and potentially compromise long-term metabolic durability.

As skeletal muscle represents a major site of glucose disposal and systemic energy expenditure [89, 90], its preservation is central to durable metabolic adaptation. Accordingly, lean mass reduction may represent a context-dependent consequence of sustained energy flux constriction, particularly when anabolic substrate availability and resistance exercise are not proportionately maintained.

### Exercise as a Secondary Metabolic Stressor

Adipose tissue dysfunction in obesity promotes the release of small extracellular vesicles enriched with oxidatively stressed mitochondrial components [66, 91], reflecting unresolved mitochondrial strain. Excessive high-intensity exercise can also provoke mitochondrial stress signaling and impaired metabolic flexibility in certain contexts [92, 93]. These observations suggest that mitochondrial stress responses can propagate systemically and may become amplified under conditions of GLP-1RA–induced chronic energy flux constriction, where nutrient inflow is reduced while oxidative demand persists.

Under such metabolically constrained states, the adaptive benefit of exercise depends on alignment between energetic stimulus and regenerative substrate availability. Exercise during GLP-1RA therapy should therefore be interpreted not solely in terms of caloric expenditure but within the broader context of substrate sufficiency and mitochondrial resilience.

## Rate-Limiting Substrate Modules Governing Adaptation Under Chronic Flux Constriction

GLP-1RA therapy imposes a sustained reduction in nutrient inflow while maintaining or redistributing oxidative throughput (Sect. 3). Under these conditions, systemic metabolic adaptation increasingly depends on the availability of substrates required to sustain redox buffering, anabolic maintenance, and enzymatic throughput. Clinical observations describing reduced protein intake, variability in lean mass trajectories, and micronutrient insufficiency during pharmacologically induced weight loss [40, 75, 94, 95] suggest that long-term metabolic stability may become sensitive to substrate availability even in the absence of overt deficiency.

Within this context, several substrate-dependent metabolic processes may function as rate-limiting determinants of physiological resilience during sustained GLP-1RA therapy (Table 1).

**Table 1:** Substrate-dependent metabolic modules under chronic energy flux constriction. Under sustained reductions in nutrient inflow during GLP-1RA therapy, substrate availability may constrain biochemical processes that support oxidative buffering, enzymatic throughput, and tissue energetic stability. Key constraint-sensitive modules include redox currency systems (NAD⁺/NADPH partitioning), amino acid–dependent redox–anabolic coupling, micronutrient-dependent enzymatic activity, and bile acid–mediated lipid absorption

Module	Biochemical role	Constraint-sensitive process	Clinical relevance	References
NAD⁺/NADPH pools	Redox currency supporting mitochondrial electron transfer, antioxidant regeneration, and biosynthetic reactions	Maintenance of oxidative buffering capacity and mitochondrial redox balance	Indicators of oxidative resilience and redox reserve during sustained FAO	[83, 96, 97]
Amino acids	Substrates for protein synthesis, glutathione production, and nitrogen balance	Coupling of antioxidant defense with anabolic maintenance of skeletal muscle and other tissues	Lean mass preservation and adequacy of protein intake during weight loss	[79, 98, 99]
Micronutrient cofactors (e.g., iron, selenium, B vitamins)	Enzymatic cofactors enabling mitochondrial respiration, antioxidant enzymes, and intermediary metabolism	Catalytic velocity of oxidative phosphorylation and metabolic pathway throughput	Micronutrient sufficiency supporting metabolic enzymatic capacity	[100–102]
Bile acids and lipid absorption pathways	Facilitation of dietary lipid and fat-soluble nutrient absorption	Maintenance of lipid assimilation and availability of fat-soluble nutrients under delayed gastric emptying	Absorptive efficiency influencing lipid-derived substrate availability	[53, 103, 104]

*FAO* fatty acid oxidation, *NAD* nicotinamide adenine dinucleotide, *NADPH* nicotinamide adenine dinucleotide phosphate (reduced form)

### NAD⁺ Partitioning as a Redox Currency Constraint

Within the flux-constrained metabolic state described above, maintenance of redox balance depends critically on the capacity of NAD⁺/NADPH-dependent regenerative systems.

Obesity-associated inflammation accelerates NAD⁺ consumption through activation of NAD⁺-utilizing enzymes such as poly(ADP-ribose) polymerase 1 (PARP-1) and cluster of differentiation 38 (CD38) [85]. Although GLP-1RAs can attenuate inflammatory tone [105], sustained reductions in nutrient inflow associated with pharmacological weight loss may influence NAD⁺ turnover kinetics in metabolically stressed tissues. Direct longitudinal human studies evaluating tissue-specific NAD⁺ dynamics during GLP-1RA therapy remain limited; therefore, current interpretations draw on evidence from obesity, aging, and caloric restriction literature. NAD⁺ can be generated through salvage, de novo, and related metabolic pathways [106], but its physiological relevance lies in dynamic partitioning between oxidized and reduced redox currencies. Conversion to NADH sustains mitochondrial electron transport, whereas NADPH supports reductive biosynthesis and glutathione-dependent antioxidant buffering [83]. Metabolic tracing studies further demonstrate that systemic NAD⁺ pools undergo inter-organ redistribution and are influenced by microbial metabolism and circulatory exchange [107, 108], indicating that NAD⁺ functions within a dynamic network rather than as a static intracellular reservoir [109]. Although described here as “pools” for conceptual simplicity, NAD⁺ and NADPH are not uniformly distributed but are frequently associated with specific enzymes or enzyme complexes, enabling localized and pathway-specific redox regulation. Accordingly, the functional impact of NAD⁺ metabolism is determined not only by absolute pool size but also by turnover flux, compartmental exchange, and redox coupling across tissues. However, increases in circulating NAD⁺ precursors do not necessarily translate into proportional expansion of tissue-specific redox capacity, as precursor metabolism and systemic NAD⁺ pools are strongly influenced by microbial conversion and circulatory metabolism [109].

Intracellular NAD⁺ metabolism is also spatially organized. Cytosolic and mitochondrial pools are functionally compartmentalized, and exchange between these domains occurs indirectly through metabolite shuttles [110]. Since mitochondrial oxidative phosphorylation depends on local NADH availability, whereas cytosolic NADPH supports reductive buffering, disproportionate redistribution between compartments may amplify localized redox imbalance even when total cellular NAD⁺ levels appear preserved. Under conditions of chronic energy flux constriction, compartment-specific regeneration efficiency may therefore become more relevant than bulk metabolite concentration. 

Within this context, a functional redox constraint may arise when oxidative throughput approaches or exceeds the regenerative capacity of NAD⁺/NADPH-dependent buffering systems. In this review, the term “*redox bottleneck*” is used as a conceptual framework describing conditions in which oxidative throughput approaches the regenerative capacity of substrate-supported NAD⁺/NADPH-dependent buffering systems under sustained energy flux constriction. Importantly, this concept does not imply overt NAD⁺ depletion; rather, it reflects disproportion between oxidative demand and the dynamic regeneration of redox currency across tissues. Accordingly, NAD⁺ availability may act as a rate-limiting metabolic currency whose adequacy is reflected by redox ratios and indicators of NAD⁺ turnover.

### Amino Acid–Dependent Antioxidant and Anabolic Coupling

Amino acid availability represents a second constraint-sensitive axis because amino acid pools support both structural maintenance and antioxidant buffering. Detoxification of lipid peroxides relies on glutathione-dependent systems, including GPX4-mediated membrane protection [111]. Since glutathione synthesis requires cysteine and related amino acids, reduced protein intake observed during GLP-1RA therapy [75] may influence both antioxidant buffering capacity and anabolic maintenance. Certain amino acids also participate in nitrogen metabolism and signaling pathways; for example, citrulline serves as a precursor for arginine synthesis and contributes to systemic nitrogen metabolism and nitric oxide signaling [98]. Under energy restriction, AMPK-mediated suppression of mTOR signaling further limits protein synthesis [52]. Clinically observed reductions in lean mass during medically induced weight loss [40] are therefore consistent with a substrate-sensitive vulnerability affecting skeletal muscle maintenance. Within this context, amino acid availability can be viewed as an integrated redox–anabolic module linking antioxidant defense with protein turnover.

Monitoring of this module may include assessment of protein intake, lean mass trajectory, and related metabolic indices reflecting nitrogen balance and oxidative status.

### Cofactor-Dependent Enzymatic Throughput

Metabolic flux is ultimately executed through enzyme systems whose catalytic activity depends on micronutrient cofactors. Magnesium supports ATP-dependent reactions; iron participates in oxygen handling and mitochondrial electron transport; selenium sustains redox-active selenoproteins; and B-complex vitamins integrate one-carbon metabolism with NAD⁺-linked pathways [100].

Obesity is frequently associated with altered mineral homeostasis, including perturbations in iron and magnesium status [14]. During GLP-1RA therapy, sustained hypophagia may further reduce effective micronutrient intake without immediately producing overt deficiency. Even modest reductions in micronutrient availability may therefore influence catalytic velocity across interconnected metabolic pathways, potentially amplifying bioenergetic and redox constraints under chronic flux limitation [112, 113].

Accordingly, micronutrient sufficiency should be considered not solely in terms of deficiency prevention but as preservation of enzymatic throughput within physiological ranges. Clinical surveillance may therefore include micronutrient status panels alongside inflammatory and metabolic indices.

### Bile Acid–Dependent Absorption

Metabolic constraint during GLP-1RA therapy may arise not only from reduced nutrient inflow but also from altered substrate handling. GLP-1RAs modify gastric emptying and can influence bile acid–related signaling and downstream intestinal transport processes [114]. Since absorption of fat-soluble vitamins and other lipid-dependent nutrients depends on bile acid–mediated mechanisms, chronic therapy may reduce effective substrate delivery even when dietary composition appears adequate. Observational studies reporting vitamin D and micronutrient insufficiencies during prolonged GLP-1RA use [94, 95, 101] suggest that nutritional monitoring may be warranted, although the magnitude and causal pathways remain to be clarified.

Altered absorption efficiency therefore represents an additional constraint-sensitive module. Its functional consequences may be reflected in the status of fat-soluble vitamins and related mineral homeostasis, particularly in individuals with pre-existing metabolic vulnerability.

Taken together, NAD⁺ partitioning, amino acid–dependent redox–anabolic coupling, cofactor-dependent enzymatic throughput, and bile acid–mediated absorption define key biochemical modules that may shape metabolic adaptation during chronic GLP-1RA–induced flux constriction. While incretin signaling improves glycemic control and inflammatory tone, sustained hypophagia shifts the locus of metabolic regulation toward substrate availability and metabolic throughput.

Within this context, adaptive stability depends on maintaining proportionality between oxidative demand and substrate-supported regenerative capacity. Disruption within these modules may progressively reduce physiological resilience during sustained pharmacological weight reduction. In this framework, the redox bottleneck represents a potential inflection point at which oxidative throughput approaches the regenerative capacity of NAD⁺/NADPH-dependent buffering systems.

## Translational Stabilization Under Chronic Flux Constriction

The translational challenge in the GLP-1RA era is not indiscriminate nutritional supplementation but preservation of substrate-sensitive metabolic control nodes that support physiological resilience during sustained pharmacological weight reduction. Under conditions of chronic hypophagia, metabolic adaptation increasingly depends on maintaining proportionality between oxidative demand and substrate-supported regenerative capacity.

**Fig. 2:**
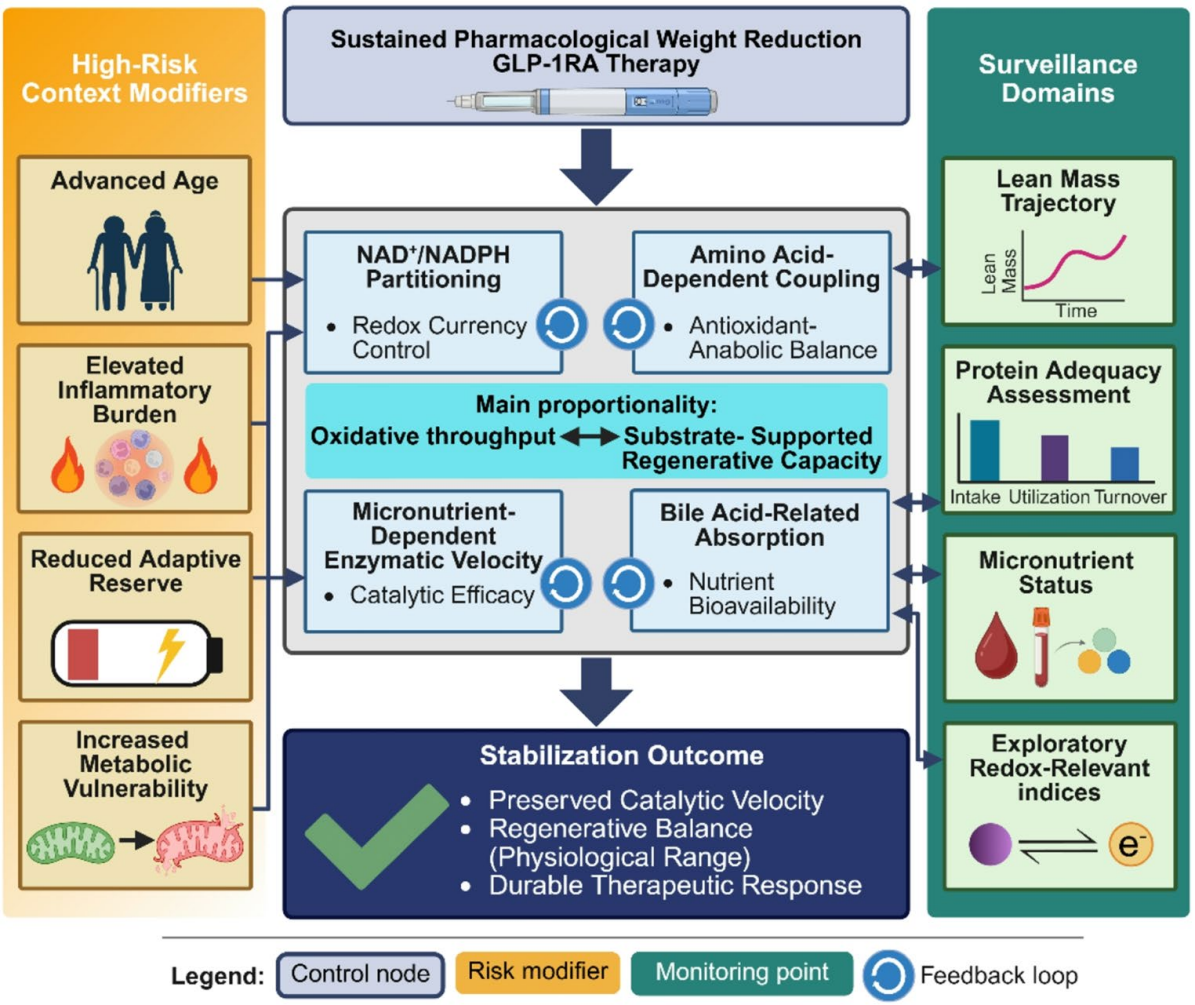
Translational control architecture for metabolic proportionality during chronic GLP-1RA therapy. Constraint-sensitive metabolic processes are operationalized as clinical control nodes to guide monitoring and stabilization during sustained pharmacological weight reduction. Surveillance domains include lean mass trajectory, protein adequacy (assessed via protein consumption [Intake], use efficiency [Utilization], and synthesis/breakdown balance [Turnover]), micronutrient status panels, and exploratory redox-relevant indices. These monitoring strategies aim to maintain proportionality between oxidative throughput and substrate-supported regenerative capacity. High-risk contexts, such as advanced age or elevated. Abbreviations: e^–^, electron; GLP-1RA, glucagon-like peptide-1 receptor agonist; NAD, nicotinamide adenine dinucleotide (oxidized form); NADPH, nicotinamide adenine dinucleotide phosphate (reduced form). Created in BioRender. YOU, J. (2026) https://BioRender.com/reskutq

Within this context, exercise stimulus should be interpreted relative to available metabolic substrates and regenerative reserve (Sect. 5). Nutritional management may therefore be more appropriately framed as stabilization of rate-limiting metabolic modules rather than post hoc correction of overt deficiency (Fig. 2).

From a clinical perspective, these metabolic considerations suggest that structured nutritional monitoring may accompany long-term GLP-1RA therapy, particularly in individuals with increased susceptibility to lean mass loss or micronutrient insufficiency. Such monitoring may include assessment of dietary protein intake, body composition trajectories, and micronutrient status in order to maintain proportionality between oxidative metabolic demand and substrate-supported regenerative capacity This perspective aligns with emerging calls to integrate pharmacologic and nutritional paradigms in obesity treatment, recognizing that durable metabolic adaptation requires coordination between drug-mediated signaling effects and substrate-dependent metabolic processes [103, 115]. 

### First Principle: Proportionality 

A defining feature of chronic energy flux constriction is that gradual shifts in substrate availability may reduce ATP/NADH generation efficiency and redox buffering reserve without exceeding conventional diagnostic thresholds. Stabilization strategies should therefore focus on maintaining proportionality between oxidative throughput and the substrate pools that support (i) redox currency, (ii) antioxidant–anabolic coupling, (iii) cofactor-dependent enzymatic velocity, and (iv) bile acid–dependent absorption (Sect. 5) This perspective also cautions against reducing GLP-1RA–associated nutrition to unsystematic supplementation. Emerging reports of lean mass changes and nutrient insufficiencies during pharmacologically induced weight loss [40, 94, 101] highlight the importance of structured monitoring rather than indiscriminate nutrient replacement.

### Monitoring as Metabolic Control

Since many relevant metabolic perturbations remain subclinical, monitoring becomes a proactive control strategy rather than a purely diagnostic measure. Observational and review-level evidence indicates that patients receiving GLP-1RAs may exhibit micronutrient insufficiencies and lean mass reduction, particularly in vulnerable populations or during prolonged therapy [94, 101]. In addition, cross-sectional dietary analyses suggest that GLP-1RA therapy can reduce nutrient intake in ways that may affect protein and micronutrient sufficiency [75]. 

Within a substrate-aware framework, clinical surveillance may therefore include periodic assessment of (i) lean mass trajectory, (ii) micronutrient status—particularly nutrients supporting enzymatic throughput and redox cycling—and (iii) fat-soluble vitamin indices as indirect indicators of bile-dependent absorption integrity.

The objective of such surveillance is to detect early metabolic drift toward constraint-sensitive states that could compromise long-term therapeutic durability. In this context, nutritional management becomes not merely supportive but an integral component of maintaining metabolic stability during pharmacological weight reduction.

### Stabilizing Anabolic Competence

Preservation of lean mass is not merely a cosmetic endpoint but a central determinant of metabolic durability during weight reduction. Emerging clinical analyses indicate that medically induced weight loss may compromise skeletal muscle unless anabolic support is appropriately maintained [40]. Human-focused reviews and meta-analyses have debated the magnitude and clinical relevance of lean mass changes during GLP-1RA therapy [41], whereas preclinical studies report context-dependent protection against muscle wasting [42, 43]. This divergence is consistent with the substrate-centered framework outlined in Sect. 5, suggesting that skeletal muscle outcomes during chronic energy restriction may depend more strongly on substrate availability than on drug signaling alone. 

Within this context, protein adequacy becomes a key control variable. Nutritional strategies should therefore emphasize sufficient protein quantity, quality, and distribution to sustain muscle protein turnover under conditions of sustained caloric restriction. 

### Redox Currency Stabilization

Maintenance of redox stability during sustained hypophagia depends on adequate NAD⁺ partitioning and glutathione-dependent buffering capacity [83]. Obesity-associated inflammation and aging can accelerate NAD⁺ turnover through PARP-1 and CD38 pathways [85, 106, 116]. Although GLP-1RAs can reduce inflammatory signaling, this effect does not necessarily ensure that precursor inflow remains proportionate to ongoing NAD⁺ turnover [75, 105]. Glutathione buffering capacity is likewise substrate-dependent, and amino acid pools supporting redox defense intersect directly with anabolic maintenance [111]. Consequently, translational strategies should emphasize preservation of substrate pools supporting redox buffering rather than indiscriminate antioxidant supplementation. 

This interpretation is consistent with clinical observations that oral glutathione supplementation has limited bioavailability, favoring approaches that maintain precursor sufficiency instead of direct replacement [117–119]. Mechanistically, the stabilization objective is to prevent progression toward a *redox bottleneck*, in which oxidative throughput approaches the regenerative capacity of NAD⁺/NADPH-dependent buffering systems.

### Stabilizing Cofactor Throughput

Micronutrients function as essential enablers of metabolic throughput rather than optional nutritional add-ons, as they determine catalytic velocity across core biochemical pathways. Obesity is frequently associated with perturbations in micronutrient status, including vulnerabilities in iron and magnesium homeostasis [14, 15, 120]. Magnesium-related literature further links these disturbances to inflammatory signaling and oxidative stress regulation [121]. During GLP-1RA–induced hypophagia, modest reductions in micronutrient intake may increase the likelihood of subclinical insufficiency. In this setting, metabolic durability may depend on maintaining cofactor availability within physiological ranges rather than pursuing aggressive replacement strategies.

Mechanistically, cofactor-dependent metabolic nodes illustrate how relatively small reductions in catalytic support can propagate across interconnected pathways involving nucleotide metabolism, redox homeostasis, and immune–metabolic regulation under conditions of metabolic stress [122–125]. Accordingly, preservation of cofactor throughput can be viewed as an integrative metabolic control point linking redox balance, anabolic maintenance, and immune function. Translationally, this perspective supports structured evaluation of micronutrient status aimed at maintaining enzymatic capacity rather than indiscriminate supplementation.

### Absorption Integrity

Stabilization strategies must account not only for nutrient intake but also for nutrient handling. Preclinical evidence indicates that liraglutide can modify bile acid profiles and gut signaling independently of feeding behavior [114]. Clinical reviews likewise suggest that GLP-1RA users may develop micronutrient insufficiencies resembling—although not identical to—patterns observed after bariatric surgery [94, 101, 102]. These observations support monitoring of fat-soluble vitamin status and related minerals as exploratory indicators of bile-dependent absorption dynamics during prolonged therapy.

Within this framework, absorption can be considered a metabolic control module: even when dietary composition appears adequate, altered gastrointestinal kinetics may reduce effective delivery of critical substrates, shifting metabolic constraint from nutrient intake to handling efficiency.

### High-Risk Contexts

Although GLP-1RA therapy is generally safe and effective, the flux-constraint framework suggests that certain populations may be more sensitive to substrate imbalance during sustained hypophagia.

**Sarcopenic obesity or advanced age (≥ 65 years)**. Age-related anabolic resistance and reduced baseline muscle reserve may increase susceptibility to lean mass loss during medically induced weight reduction [40]. Clinical concern regarding skeletal muscle changes during GLP-1RA therapy remains debated [40], and preclinical studies have reported context-dependent protection against muscle wasting in selected settings [42, 43]. These findings indicate that baseline muscle reserve may influence adaptive stability under chronic energy flux constriction. 

**Individuals with pre-existing micronutrient insufficiency.** Obesity is frequently associated with perturbations in micronutrient status, including vulnerabilities in iron and magnesium homeostasis [14, 15, 120]. Observational and review-level evidence also suggests that micronutrient insufficiencies may occur during prolonged GLP-1RA therapy [94, 95, 101]. In individuals with marginal baseline status, reduced nutrient intake and altered absorption kinetics may increase susceptibility to enzymatic throughput constraints. 

**Type 2 diabetes with chronic kidney disease or elevated inflammatory burden**. Obesity-associated inflammation can accelerate NAD⁺ turnover through PARP-1 and CD38 pathways [85, 97, 116]. Although GLP-1RAs attenuate inflammatory signaling [105], NAD⁺ metabolism remains dynamically regulated and tissue-specific [83, 106, 109]. In metabolically stressed populations, regenerative capacity may therefore be more sensitive to sustained substrate limitation.

**Postmenopausal women or individuals at risk for bone–muscle interaction decline.** Observational studies suggest that GLP-1RA therapy may be associated with changes in musculoskeletal parameters under conditions of rapid weight loss [34, 40]. Combined anabolic and micronutrient vulnerabilities in these populations may influence musculoskeletal resilience.

These groups do not represent contraindications for GLP-1RA therapy. Rather, they illustrate clinical contexts in which substrate-sensitive proportionality may be more vulnerable during sustained hypophagia.

### Operationalizing Surveillance

Within the flux-constriction framework, monitoring is conceptualized as preservation of metabolic proportionality rather than detection of overt deficiency.

**Lean Mass Trajectory.** Medically induced weight loss can influence skeletal muscle mass [40]. Reviews and meta-analyses report variable lean mass outcomes during GLP-1RA therapy. Periodic body composition assessment—where feasible—may therefore help contextualize total weight reduction relative to lean mass preservation.

**Redox-Relevant Indices (Exploratory).** NAD⁺ metabolism integrates mitochondrial energy production with antioxidant buffering [83]. Obesity and aging are associated with altered NAD⁺/Sirtuin 1 (SIRT1)/PARP dynamics [85]. Although routine NAD⁺ quantification is not currently part of clinical practice, longitudinal incorporation of redox-related biomarkers in research settings may help determine whether flux–regeneration proportionality remains stable during prolonged therap.

**Micronutrient Throughput.** Iron, magnesium, selenium, and B-complex vitamins support enzymatic velocity and mitochondrial metabolic throughput [100]. Given the association between obesity and micronutrient perturbations [14] and reports of insufficiencies among GLP-1RA users [101], periodic evaluation of micronutrient status may be appropriate in higher-risk populations.

**Protein Sufficiency.** GLP-1RA therapy frequently reduces overall energy intake and may lower protein consumption [75]. Since amino acid availability supports both anabolic signaling [52] and glutathione-dependent antioxidant buffering [111], structured dietary assessment may help preserve anabolic competence. This proportionality-based approach prioritizes early identification of constraint-sensitive drift rather than indiscriminate supplementation.

**Exercise Prescription.** Exercise remains a cornerstone of obesity management, and combined exercise with GLP-1RA therapy can support weight maintenance [49]. However, under sustained energy flux constriction, excessive training volume without nutritional alignment may increase mitochondrial stress signaling [92, 93]. Exercise stimulus should therefore be interpreted relative to substrate-supported regenerative capacity rather than caloric expenditure alone. Resistance training may preferentially support anabolic competence, whereas abrupt escalation of high-intensity endurance training could increase oxidative liability in selected contexts.

Taken together, nutritional management during GLP-1RA therapy may be viewed as stabilization of substrate-sensitive metabolic control nodes operating under chronic flux constriction. The central question is whether proportional maintenance across redox currency, anabolic coupling, cofactor throughput, and absorption integrity preserves tissue resilience during sustained therapy. This framework generates testable predictions and potential monitoring endpoints—including lean mass trajectory, micronutrient status, fat-soluble vitamin surveillance, and exploratory redox-related biomarkers—that can be evaluated in future longitudinal and interventional studies. 

## Discussion and Clinical Perspectives

GLP-1 receptor agonists have transformed obesity therapy by enabling sustained and clinically meaningful weight reduction. However, their biological effects extend beyond appetite suppression. By persistently reducing nutrient inflow while maintaining or redistributing oxidative demand, GLP-1RA therapy establishes a state of chronic energy flux constriction. Within this context, metabolic stability may depend on proportionality between substrate availability and pharmacologically induced metabolic demand. This perspective complements incretin-centered therapeutic frameworks by emphasizing metabolic flux proportionality as a determinant of long-term physiological resilience during pharmacological weight reduction. 

The perspective outlined here emphasizes regenerative capacity relative to metabolic flux rather than absolute accumulation of reactive oxygen species. Oxidative throughput can approach or exceed regenerative capacity without requiring overt depletion of metabolites. Evidence that systemic NAD⁺ pools undergo inter-organ redistribution and dynamic turnover [96, 107, 126] supports the view that redox stability reflects flux kinetics rather than static metabolite concentrations. Under sustained hypophagia, disproportion between oxidative demand and regenerative efficiency may therefore emerge as a functional constraint even in the absence of classical deficiency states.

From this perspective, receptor-level signaling alone may be insufficient to ensure durable tissue-level outcomes. Under conditions of reduced nutrient inflow, therapeutic durability may depend on whether substrate-sensitive modules—including NAD⁺/NADPH redox currency, amino acid–dependent antioxidant–anabolic coupling, micronutrient-supported catalytic velocity, and mitochondrial buffering systems—remain proportionate to oxidative demand. Importantly, this framework does not assume universal deficiency during GLP-1RA therapy; rather, it predicts heightened sensitivity to substrate imbalance during sustained pharmacological hypophagia. Subclinical reductions in protein intake, micronutrient availability, or redox precursor turnover may therefore influence long-term metabolic resilience even without overt deficiency syndromes.

This model also generates testable predictions. Prospective studies integrating body composition trajectories, structured dietary assessment, and redox-relevant biomarkers during GLP-1RA therapy may clarify whether preservation of rate-limiting substrate modules supports lean mass maintenance and metabolic stability. Stratified analyses in higher-risk groups—such as older adults, individuals with sarcopenic obesity, or those with elevated inflammatory burden—may further determine whether adaptive thresholds differ across metabolic contexts.

Several uncertainties remain. It is not yet clear whether the proposed metabolic constraints are specific to GLP-1RA therapy or represent a broader feature of sustained caloric restriction and weight loss [49, 50]. While GLP-1RAs uniquely combine pharmacological signaling with reduced nutrient inflow, similar redox constraints may arise in other contexts, including bariatric surgery or intensive dietary interventions. In addition, although changes in oxidative stress markers have been reported, direct evidence linking GLP-1RA therapy to constrained redox buffering capacity remains limited.

Overall, GLP-1RA therapy represents sustained remodeling of systemic energy dynamics under conditions of chronically reduced nutrient inflow. Within this flux-constrained state, long-term metabolic resilience likely reflects the interaction between receptor-mediated signaling and substrate-supported regeneration. Observations of variable lean mass outcomes and dietary shifts during GLP-1RA therapy [40, 75] suggest that substrate availability may influence therapeutic durability even in the absence of overt deficiency. Accordingly, integration of structured nutritional surveillance with pharmacological therapy may represent an important component of long-term GLP-1RA management. 

Taken together, the flux-constraint perspective reframes GLP-1RA therapy as a systemic metabolic state in which therapeutic durability depends on maintaining proportionality between oxidative throughput and substrate-supported regenerative capacity.

## Conclusion

GLP-1RA therapy induces sustained receptor signaling while simultaneously reducing nutrient inflow, generating a systemic metabolic state characterized by chronic energy flux constriction. Under these conditions, metabolism shifts toward increased reliance on lipid-derived substrates and elevated oxidative throughput. When oxidative demand approaches or exceeds the regenerative capacity of NAD^+^/NADPH-dependent buffering systems, a conditional rate-limiting state—conceptualized here as a *redox bottleneck*—may emerge.

This framework highlights that long-term metabolic stability during GLP-1RA therapy depends not only on energy balance but also on maintaining proportionality between oxidative throughput and substrate-supported regenerative capacity. Several metabolic nodes appear particularly sensitive to this constraint, including NAD^+^ partitioning, amino acid-dependent antioxidant-anabolic coupling, micronutrient-dependent enzymatic activity, and bile acid-mediated nutrient absorption. 

Future research should focus on defining biomarkers that reflect flux proportionality and redox reserve in patients undergoing GLP-1RA therapy. Such approaches may help refine nutritional strategies and improve the long-term metabolic durability of pharmacological obesity treatment.

## Key References

- Aronne LJ, Horn DB, le Roux CW, Ho W, Falcon BL, Gomez Valderas E, et al. Tirzepatide as Compared with Semaglutide for the Treatment of Obesity. *N Engl J Med*. 2025;393(1):26–36. doi: 10.1056/NEJMoa2416394. (Of outstanding importance).
  - ○ This head-to-head randomized trial directly compared tirzepatide with semaglutide for obesity treatment and provides high-level evidence for the magnitude of weight loss achievable with current incretin-based pharmacotherapy. It establishes the clinical context in which downstream metabolic adaptation, altered nutrient intake, and long-term physiological tradeoffs should be interpreted.

- Xie Y, Choi T, Al-Aly Z. Mapping the effectiveness and risks of GLP-1 receptor agonists. *Nat Med*. 2025;31(3):951 − 62. doi: 10.1038/s41591-024-03412-w. (Of outstanding importance).
  - ○ This large-scale real-world study mapped both beneficial and adverse outcomes associated with GLP-1 receptor agonist therapy across multiple organ systems. It is particularly important because it supports the view that GLP-1RA treatment should be understood as a systemic metabolic intervention rather than a weight-loss effect alone.

- Johnson B, Milstead M, Thomas O, McGlasson T, Green L, Kreider R, et al. Investigating nutrient intake during use of glucagon-like peptide-1 receptor agonist: a cross-sectional study. *Front Nutr*. 2025;12:1566498. doi: 10.3389/fnut.2025.1566498. (Of importance)
  - ○ This cross-sectional study provides direct evidence that GLP-1 receptor agonist use may be accompanied by reduced intake of protein and selected micronutrients. These findings are important because they support the manuscript’s central argument that appetite suppression can constrain the nutritional substrate supply required for durable metabolic adaptation.

- Scott Butsch W, Sulo S, Chang AT, Kim JA, Kerr KW, Williams DR, et al. Nutritional deficiencies and muscle loss in adults with type 2 diabetes using GLP-1 receptor agonists: A retrospective observational study. *Obes Pillars*. 2025;15:100186. doi: 10.1016/j.obpill.2025.100186. (Of importance).
  - ○ This retrospective observational study links GLP-1 receptor agonist therapy with nutritional deficiencies and muscle loss in adults with type 2 diabetes. It is important because it extends concern beyond weight reduction itself to the quality of weight loss and the need for structured nutritional and body-composition monitoring.

- Lincoff AM, Brown-Frandsen K, Colhoun HM, Deanfield J, Emerson SS, Esbjerg S, et al. Semaglutide and Cardiovascular Outcomes in Obesity without Diabetes. *N Engl J Med*. 2023;389(24):2221-32. doi: 10.1056/NEJMoa2307563. (Of importance).
  - ○ This cardiovascular outcomes trial demonstrated that semaglutide reduces major adverse cardiovascular events in people with obesity without diabetes. The study is important because it confirms that GLP-1RA therapy has clinically meaningful systemic effects, reinforcing the need to understand how metabolic benefit is integrated with broader nutritional and physiological adaptation.

- Chen S, He M, Qin Y, Tian J, Liang Z, Li Y, et al. Effects of 3-month liraglutide treatment on oxidative stress and inflammation in type 2 diabetes patients with different urinary albumin-to-creatinine ratio categories. *Medicine (Baltimore)*. 2024;103(47):e40438. doi: 10.1097/MD.0000000000040438. (Of importance).
  - ○ This clinical study evaluated changes in oxidative stress and inflammatory markers after liraglutide treatment in patients with type 2 diabetes. It is important because it provides human biomarker-level support for the idea that GLP-1RA therapy influences redox-related physiology in parallel with its metabolic actions.

## Data Availability

No datasets were generated or analysed during the current study.
